# Enhanced Immunogenicity of a Whole-Inactivated Influenza A Virus Vaccine Using Optimised Irradiation Conditions

**DOI:** 10.3389/fimmu.2021.761632

**Published:** 2021-11-24

**Authors:** Eve Victoria Singleton, Chloe Jayne Gates, Shannon Christa David, Timothy Raymond Hirst, Justin Bryan Davies, Mohammed Alsharifi

**Affiliations:** ^1^ Research Centre for Infectious Diseases, Department of Molecular and Biomedical Sciences, University of Adelaide, Adelaide, SA, Australia; ^2^ Gamma Vaccines Pty Ltd, Yarralumla, ACT, Australia; ^3^ Irradiations Group, Australian Nuclear Science and Technology Organisation, Lucas Heights, NSW, Australia

**Keywords:** influenza A virus, gamma radiation, vaccine, sterility assurance level (SAL), irradiation conditions, universal influenza A vaccine

## Abstract

Influenza A virus presents a constant pandemic threat due to the mutagenic nature of the virus and the inadequacy of current vaccines to protect against emerging strains. We have developed a whole-inactivated influenza vaccine using γ-irradiation (γ-Flu) that can protect against both vaccine-included strains as well as emerging pandemic strains. γ-irradiation is a widely used inactivation method and several γ-irradiated vaccines are currently in clinical or pre-clinical testing. To enhance vaccine efficacy, irradiation conditions should be carefully considered, particularly irradiation temperature. Specifically, while more damage to virus structure is expected when using higher irradiation temperatures, reduced radiation doses will be required to achieve sterility. In this study, we compared immunogenicity of γ-Flu irradiated at room temperature, chilled on ice or frozen on dry ice using different doses of γ-irradiation to meet internationally accepted sterility assurance levels. We found that, when irradiating at sterilising doses, the structural integrity and vaccine efficacy were well maintained in all preparations regardless of irradiation temperature. In fact, using a higher temperature and lower radiation dose appeared to induce higher neutralising antibody responses and more effective cytotoxic T cell responses. This outcome is expected to simplify irradiation protocols for manufacturing of highly effective irradiated vaccines.

## Introduction

Influenza A virus (IAV) is a major health concern and causes significant morbidity and mortality on a global scale. The most at-risk groups for development of serious IAV symptoms or secondary complications are infants, the elderly, the immunocompromised, and pregnant women (1). Vaccination remains the most effective method to combat IAV infection, though current inactivated vaccines have major valency and efficacy limitations. Existing formulations consist of purified IAV surface proteins haemagglutinin (HA) and neuraminidase (NA) of 2 IAV strains and an additional 1 or 2 influenza B virus strains predicted to circulate in a given year. Whilst effective at protecting against ‘vaccine-included’ strains, the immune responses induced by current IAV vaccines are antibody-based only and provide minimal protection against strains not included in a given formulation (i.e. non-vaccine strains). In general, current IAV vaccines are ineffective against newly emerging seasonal strains and novel pandemic strains and must also be updated and redistributed every year due to the highly mutagenic nature of IAV surface proteins.

In order to increase vaccine coverage and minimise IAV-related morbidity and economic costs, new cross-protective IAV vaccines must be developed. Our group has previously demonstrated that a gamma (γ)-irradiated whole-inactivated IAV vaccine (γ-Flu) has the ability to induce cross-protective responses against vaccine-included and non-included strains (2). Our previous publications illustrated that mice vaccinated with a single dose of γ-Flu (consisting of a H1N1 strain) were able to survive a lethal dose of a non-vaccine H1N1 strain (drifted), a heterosubtypic H3N2 strain (3), and the highly pathogenic avian H5N1 (4). The ability of γ-Flu to induce cross-protective immunity is specifically due to induction of cytotoxic T-cell responses against conserved internal IAV proteins (5).

In addition to our work on the development of γ-Flu (6, 7), several vaccines using γ-irradiation are currently in clinical trials including vaccines against human immunodeficiency virus (8) and malaria (9, 10). Given these promising results, it is crucial to determine the optimal conditions to ensure both sterility and high immunogenicity of γ-irradiated vaccines. Importantly, all γ-irradiated products intended to come into contact with human tissue must meet the internationally accepted sterility assurance level (SAL) of 10^-6^, or a one in a million chance that an infectious unit escapes sterilisation (11). In general, while the sterilising dose required to achieve an acceptable Sterility Assurance Leve (DS_SAL_) is dependent upon starting titre, it is heavily influenced by environmental conditions, particularly irradiation temperature (12). For example, viruses irradiated at lower temperatures (e.g. whilst frozen) are expected to be more resistant to irradiation damage. It is well-established that γ-irradiation causes damage to pathogens *via* two mechanisms, termed the direct and indirect effects. The slower inactivation of frozen materials is due to reduced indirect effects, as the production and movement of damaging free radicals is physically restricted (13, 14). This preserves antigenic epitopes within vaccine preparations (15, 16), but requires increased irradiation doses to achieve sterility. Large-scale irradiation of vaccine materials whilst maintained in a frozen state is likely to pose feasibility issues. Conversely, adopting a higher irradiation temperature (e.g. room temperature) increases viral sensitivity to irradiation damage, resulting in a much lower sterilising doses and faster irradiation time (12). This should increase the practicality of inactivation methods when scaled-up for manufacturing, particularly if vaccine immunogenicity is maintained. However, while faster inactivation at higher temperatures is desirable for most irradiated products (e.g. medical items, foods, etc.), the immunogenicity of vaccines treated in this manner is expected to be reduced due to amplification of indirect effects (17–19). Thus, an appropriate balance between sterilisation requirements and vaccine antigenicity should be assessed. In fact, previous studies did not address vaccine efficacy after irradiating with different doses that achieve the SAL at different irradiation temperatures.

In this study, we calculated the DS_SAL_ for γ-Flu irradiated on dry ice (DI), ice or at room-temperature (RT). We subsequently assessed structural integrity and vaccine efficacy of these three preparations in animal models. Our data show that vaccine efficacy is well maintained when irradiating at higher temperatures using lower doses of sterilising radiation. This could potentially open an avenue to use lower radiation doses to reduce manufacturing time and costs, while suitably maintaining both sterility and vaccine immunogenicity.

## Materials and Methods

### Ethics Statement

This study was conducted in compliance with the *Australian Code of Practice for the Care and Use of Animals for Scientific Purposes* (20). These studies were approved by the University of Adelaide Animal Ethics Committee under ethics approval number S-2018-013.

### Virus Stocks

Influenza A/Puerto Rico/8/1934 [H1N1] (A/PR8) and A/California/07/2009 [H1N1] (A/California) were grown in the allantoic cavity of 10-day-old embryonated chicken eggs at 37°C for 48 hours. Eggs were then chilled at 4°C overnight, and infected allantoic fluid was harvested and clarified by centrifugation at 3272 × g for 10 minutes.

Vaccine concentration and purification was performed by haemadsorption as described previously (21). Briefly, infected allantoic fluid was incubated with chicken erythrocytes at 4°C for 1.5 hours to allow virus adsorption to red blood cells (RBCs). Samples were then centrifuged to pellet virus-RBC complexes, and allantoic fluid supernatant was removed. Pellets were resuspended in 0.85% saline and incubated at 37°C for 1.5 hours to allow virus release from RBCs. Samples were then centrifuged to pellet RBCs, and the virus-containing supernatant was collected, aliquoted and stored at -80°C until required. Titres of concentrated IAV stocks were estimated as 3 × 10^9^ TCID_50_/mL and 4 × 10^7^ TCID_50_/mL for A/PR8 and A/California, respectively, by TCID_50_ assay.

### Gamma Irradiation of IAV Vaccines

Concentrated IAV stocks of A/PR8 at a TCID_50_ of 3 × 10^9^ TCID_50_/mL were inactivated by γ-irradiation at the following temperature conditions: frozen on dry-ice (DI, approximately -78.5°C), cold on ice water (ice, 4-8°C) or at room temperature (RT, 24-27°C), generating DI-γ-Flu, Ice-γ-Flu, and RT-γ-Flu respectively. Sterilising doses were calculated as described previously to be 35 kGy for DI-γ-Flu and 16 kGy for Ice- and RT-γ-Flu (12).

Irradiation was performed using a cobalt-60 batch-type gamma irradiator at the Australian Nuclear Science and Technology Organisation (ANSTO, NSW). Samples for irradiation were double-contained in cryovials within 10 ml falcon tubes and placed in a 45 litre cooler box, sited in a fixed, reproducible location within the irradiation room. Samples were placed in pre-determined positions in the cooler box at various distances from the radiation source so that multiple doses could be delivered simultaneously. The cooler box was then filled with water (RT), chilled water containing ice blocks, or powdered dry ice for the different temperature conditions. Radiation doses were measured using calibrated Fricke (22) and ceric-cerous dosimeters (23) and dose rates varied from 0.3-1.6 kGy/h.

Temperature was monitored with a calibrated digital temperature probe connected to a data logger (Novus LogBox-AA) for ice and RT samples for the duration of irradiation, and non-irradiated control samples were subject to the same temperature conditions, stored out of the irradiation room. After irradiation, all samples were stored at -80°C until required.

### Virus Titrations

IAV was titrated by 50% tissue culture infectious dose (TCID_50_) assay using Madin-Darby canine kidney (MDCK) cells. MDCK cells were maintained in Dulbecco’s Modified Eagle’s Medium (DMEM) with 10% foetal bovine serum (FBS) and 1% penicillin/streptomycin (P/S). MDCK cells were kept at 37°C with 5% CO_2_ and were passaged with trypsin when they reached approximately 90% confluence. For TCID_50_ assay, MDCK cells were seeded in 96-well round-bottomed plates at 5 × 10^4^ cells/well. After 24h incubation, confluent cell monolayers were infected with 10-fold serial dilutions of IAV in DMEM supplemented with 8% trypsin for virus activation. Plates were incubated at 37°C for 3 days, then amplified virus in culture supernatants was detected by the addition of 0.6% packed RBCs based on pellet or mesh formation, with a mesh being considered positive for IAV. 50% infectious doses (TCID_50_/mL) were calculated using the Reed and Muench method (24).

For haemagglutination assays, serial dilutions of IAV were performed in 0.85% saline in a 96-well round-bottomed microtitre plate. 0.6% packed RBCs in 50μL were added to each well and plates were scored for mesh or pellet formation. The reciprocal of the highest virus dilution showing a mesh was used to determine the total haemagglutination units (HAU/mL).

Sterility testing was also performed after γ-irradiation of A/PR8 to ensure that the doses selected were sterile. MDCK cells were plated in 96-well flat-bottomed microtitre plates at 2 × 10^4^ cells/well. γ-Flu was activated with 10μg/mL TPCK-trypsin at 37°C for 30 minutes then diluted 1:10 in DMEM + 1% P/S + 0.5μg/mL TPCK-trypsin. Inoculum was added to MDCK cells at an MOI-equivalent of 600 and cells were then incubated at 37°C for 24 hours to allow virus replication (passage 1). Supernatant was then collected and used to infect fresh MDCK monolayers (passage 2). This was then repeated for passage 3. At the time of collecting supernatant, cells were washed with PBS then fixed and permeabilised with 1:1 acetone:methanol (v/v) at 4°C for 15 minutes. Cells were then stained with polyclonal mouse anti-A/PR8 serum (1:200 dilution in PBS) for 1 hour at 4°C followed by Alexa-fluor^®^ 488 goat anti-mouse IgG secondary antibody (Life Technologies, 1:500 dilution). DAPI was used to stain cell nuclei (1μg/mL in DAPI). Images were taken using the Nikon TiE inverted fluorescence microscope and analysed using NIS elements software (Tokyo, Japan).

### Neuraminidase Assay

Two-fold serial dilutions of live and irradiated IAV samples were performed in PBS in triplicate. Samples were then incubated with 0.125mM of 2’-(4-Methylumbelliferyl)-α-D-N-acetylneuraminic acid (4-MUNANA, Sigma M8639) at 37°C for 1 hour, facilitating cleavage of 4-MUNANA by active IAV neuraminidase (NA) into the fluorescent substrate 4-Methylumbelliferyl (4-MU). 4-MU (Sigma M1381) was also included at increasing concentrations to generate standard curves. After 1 hour the assay was stopped with ice-cold 0.5M Na_2_CO_3_ at pH 10.5 and read using a SpectraMax fluorescent plate reader with an excitation wavelength of 365nm and emission wavelength of 450nm.

### Transmission Electron Microscopy

Irradiated IAV at different temperatures was loaded onto 3mm formvar/carbon coated grids (approx. 3 μL/grid) and left to settle for 3 to 5 minutes. Grids were blotted to dry, washed, then stained with 2% uranyl acetate for 3 minutes. Grids were then washed with PBS and blotted to dry prior to visualisation using the FEI Tecnai G2 Spirit TEM (Adelaide Microscopy, University of Adelaide).

### Mice

6-8 week old female BALB/c mice were vaccinated intranasally under ketamine anaesthetic (10% ketamine, 1% xylazil in sterile water, inject IP at 10μL/gram of body weight) with 32μL of either PBS (mock-vaccine control) or A/PR8-derived γ-Flu irradiated at different temperatures (9.6 × 10^7^ TCID_50_-equivalent/mouse). Immune serum was collected 20 days post-immunisation by submandibular bleeding. Mice were then challenged intranasally with lethal IAV on day 21 (3 weeks post-immunisation), under ketamine anaesthetic as above. Lethal doses were determined by challenging mice with serially diluted IAV. The lowest virus concentration that gave 100% lethality in mice was selected (data not shown). Challenge doses used were 1.6 × 10^2^ TCID_50_/mouse for A/PR8 and 1.3 × 10^5^ TCID_50_/mouse for the human isolate A/California. A higher dose was required to achieve lethality for A/California. Weight loss was measured daily for a period of 21 days post-challenge, with a 20% loss of starting body weight was used as a humane end point.

### Antibody Responses

Enzyme-linked immunosorbent assay (ELISA) was used to measure IgG responses in serum samples from vaccinated and control mice. Plates were coated with A/PR8 in bicarbonate/carbonate coating buffer and incubated overnight at room temperature. Plates were then blocked with 2% skim milk for 2 hours. Serum samples were serially diluted then added to the plate for 2 hours at room temperature. Plates were washed and horseradish peroxidase-conjugated goat anti-mouse IgG antibody (1:10,000 dilution in blocking buffer, Thermo Scientific) was added to each well. After 2 hours at room temperature, plates were washed, and colour was developed using TMB peroxidase substrate in the dark for 30 minutes then the reaction was stopped with 2M H_2_SO_4_. Absorbance was measured at 450nm using a Bio-Tek Instruments plate reader. The reciprocal of the highest dilution to give absorbance readings higher than naïve mice + 3 standard deviations was considered the IgG titre.

To measure neutralising antibody responses, a focus-forming inhibition assay was used. Monolayers of MDCK cells were treated with 0.1 MOI of A/PR8 that has been pre-incubated with serial dilutions of immune serum. Virus was allowed 2 hours to adhere to cells then inoculum was removed, and cells were washed with PBS. Fresh media was added, and cells were incubated at 37°C for a further 22 hours. Staining procedure and visualisation were performed as described for sterility testing. For measuring A/California neutralisation, the primary antibody used was polyclonal murine anti-A/California serum (1:200 dilution). Secondary antibody was Alexa-fluor^®^ 488 goat anti-mouse IgG secondary antibody (Life Technologies, 1:500 dilution).

### Cytotoxic T Lymphocyte Assay

Cytotoxic T lymphocyte (CTL) assays were performed as described previously (15). Mice were vaccinated intravenously with 3 × 10^8^ TCID_50_-equivalent of γ-Flu. 7 days later, spleens were harvested from naïve donor mice, minced, and pushed through a 70μm filter to generate a single-cell suspension. Cells were then split into equal populations, and one was pulsed with K^d^-restricted influenza nucleoprotein (NP) peptide (NPP, sequence: TYQRTRALV) and stained with CFSE (NPP-Pulsed). The second population was stained with cell tracker red (CTR) only (Unpulsed). The cells were mixed at a 1:1 ratio and injected intravenously into vaccinated and non-vaccinated control mice at 10^7^ cells/mouse. 24 hours later, all mice were sacrificed, and spleens were harvested and processed into a single-cell suspension prior to fixing using 1% PFA. Labelled pulsed and non-pulsed cells were acquired using the LSRII flow cytometer (BD Biosciences), and data was analysed using FlowJo software (Treestar Incorporated).

### Statistical Analysis

Statistical analysis was performed using GraphPad Prism version 8 (GraphPad Software, La Jolla, CA, USA). Quantitative results were expressed as mean ± SEM. One-way ANOVA (with Tukey’s multiple comparisons test) was used for comparison of data from 3 or more groups. Survival data were analysed using Fisher’s exact test (two-tailed). P-values < 0.05 (95% confidence) were considered statistically significant.

## Results

### Structural Integrity of γ-Flu

**Figure 1:**
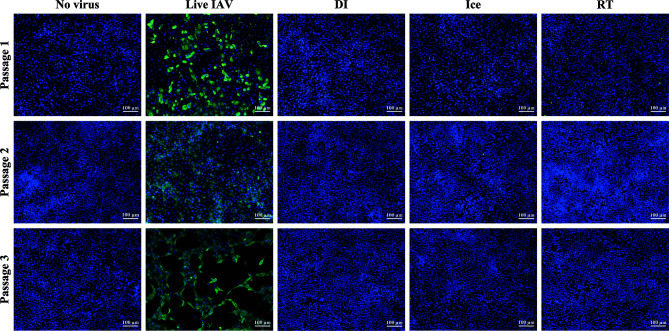
Sterility of γ-irradiated IAV. Sterility of γ-Flu was assessed by multiple passages in MDCK cells. Live A/PR8 or no virus were used as controls. γ-Flu was added to cells at an MOI equivalent of 600. Supernatant from passage 1 was collected 24 hours later and used to infect monolayers of MDCK cells for passage 2, this was then repeated for passage 3. Cell monolayers were stained with DAPI (blue), and IAV-positive cells were visualised with FITC-fluorescence (green). Samples were tested in triplicate and representative images are presented for each group at each passage.

The aim of this study was to compare immunogenicity of vaccines irradiated to the SAL across different temperatures. Sterilising doses required to reduce virus titre to an acceptable SAL of 10^-6^ were calculated as described previously (12). For DI-irradiation, the DS_SAL_ was determined to be 35 kGy (DI-γ-Flu) and for ice- and RT-irradiation the sterilising dose was calculated to be 16 kGy (Ice-γ-Flu, RT-γ-Flu). Sterility testing based on multiple *in vitro* passages was performed to ensure complete inactivation of irradiated materials. Live and irradiated IAV samples were passaged three times in MDCK cells, with supernatants from each treated monolayer (or passage) used to treat the next MDCK monolayer. After 3 passages, monolayers were fixed and stained for IAV infection. No virus infectivity was detected in any of the MDCK monolayers treated with irradiated preparations for all 3 passages, whereas replication of live virus was amplified at each passage (**Figure 1**). The irradiated materials were thus confirmed to be sterile and appropriate for subsequent *in vitro* and *in vivo* experiments.

**Figure 2:**
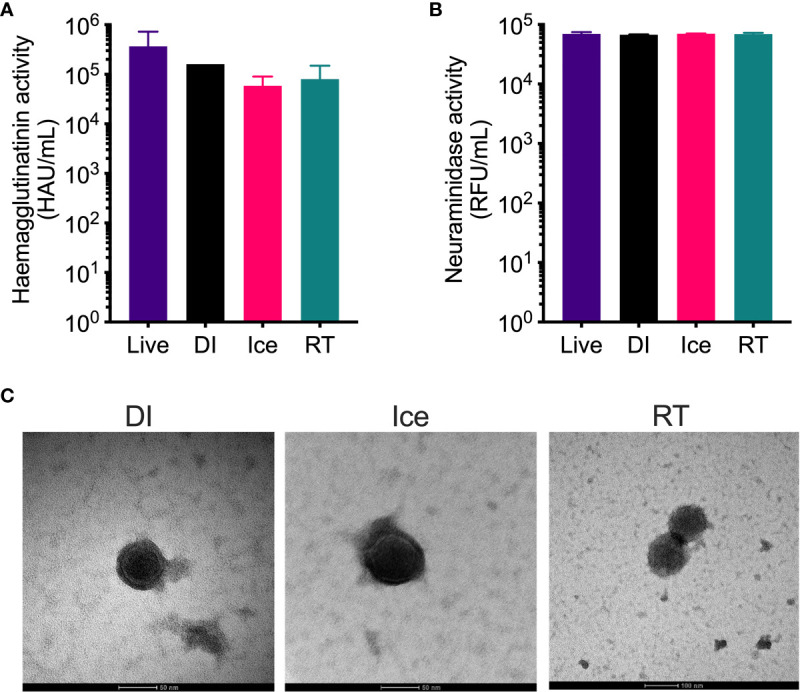
Structural integrity of IAV is maintained after γ-irradiation at different temperatures. γ-Flu preparations were inactivated with either: 16 kGy at RT, 16 kGy on ice, or 35 kGy on DI. Structural integrity of these preparations was then assessed by **(A)** haemagglutination assay, **(B)** neuraminidase assay and **(C)** transmission electron microscopy. Quantitative data is expressed as mean ± SEM (n = 3). Data is analysed by one-way ANOVA and results were not significant.

The structural integrity of the IAV within each vaccine preparation was then assessed by HA and NA functionality assays. While hemagglutination assay show reduced HA activity for all γ-Flu preparations compared to live IAV (**Figure 2A**), no significant difference was detected between the three irradiated samples despite the highly varied temperature conditions used for irradiation. Furthermore, **Figure 2B** demonstrates that the functionality of NA proteins in each γ-Flu preparation was not affected by irradiation, with all three vaccine formulations showing comparable NA enzymatic activity to live IAV. Transmission electron microscopy was then used to examine whole virion structure. Representative images in **Figure 2C** show that virions within all three irradiated preparations were intact and retained spherical IAV structure. This shows that in addition to having minimal impact on surface proteins, exposing IAV to DS_SAL_ at relative temperature conditions does not cause substantial damage to viral envelopes.

### Efficacy of γ-Flu in Mice

**Figure 3:**
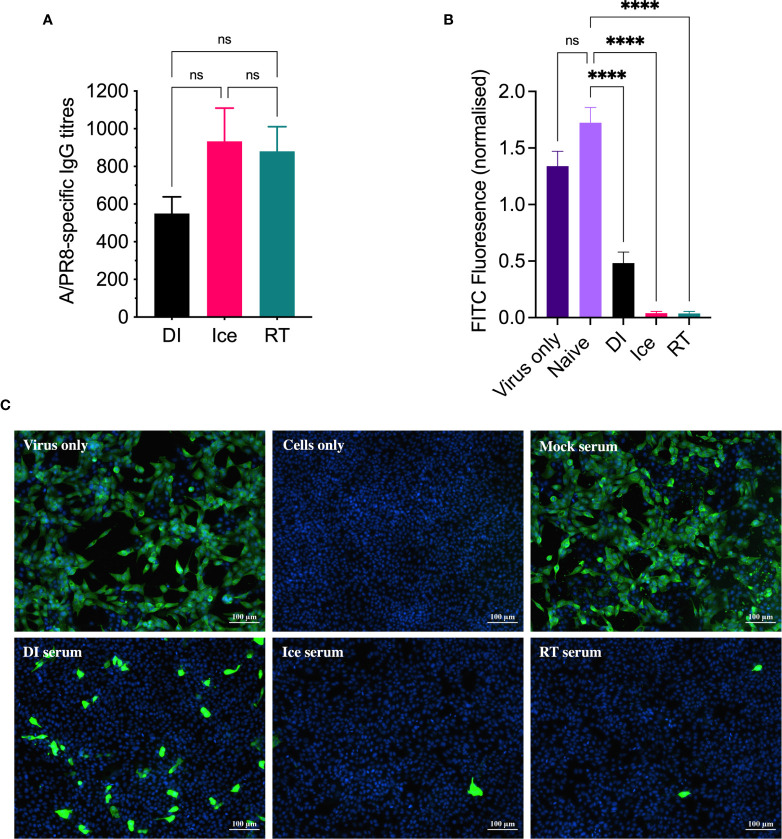
DI-γ-Flu induces reduced neutralising antibody responses when compared to Ice- and RT-γ-Flu. Mice were vaccinated intranasally with DI-γ-Flu, Ice-γ-Flu, RT-γ-Flu or PBS. Serum samples were collected 20 days post-vaccination. **(A)** IgG responses were measured by direct ELISA. Data is collated from two independent experiments (n = 5 mice per repeat) and analysed by One-Way ANOVA (not significant difference). **(B)** Neutralising antibody responses were measured by FFI. Live virus was treated with pooled naïve serum or pooled immune serum from vaccinated mice (n = 10 serum samples pooled within each vaccine group), then virus:serum mixtures were used to infect MDCK cell monolayers at MOI = 0.1. Each virus:serum mixture was tested in triplicate. FITC-fluorescence was quantified as an indicator of IAV infection and was normalised using the corresponding DAPI-fluorescence in each well (indicates the number of cell-nuclei). Data presented as mean FITC fluorescence ± SEM and analysed by one-way ANOVA (****p < 0.0001, ns, no significance). **(C)** Representative images from FFI assay showing IAV infectivity levels after treatment with pooled naïve and immune serum at a 1:10 dilution.

Given that all three γ-Flu preparations appeared suitably intact in terms of virion structure and protein functionality, we next assessed their efficacy as vaccine candidates in animal models. Initially, mice were vaccinated intranasally with a single dose of each vaccine preparation (DI-γ-Flu, Ice-γ-Flu, or RT-γ-Flu), or with PBS as a mock-vaccine control. 20 days post-immunisation, sera was harvested from all animals and an ELISA was performed to determine IAV-specific IgG titres. As shown in **Figure 3A**, all three γ-Flu preparations induced strong IgG responses above PBS-mock control levels, and no significant difference was detected between IgG titres induced by the three γ-Flu preparations. Interestingly, whilst not significant, there was a trend towards lower IgG responses detected in serum samples from mice vaccinated with DI-γ-Flu.

Following this, a focus-forming inhibition assay was performed to determine the ability of γ-Flu-induced antibodies to inhibit receptor binding and IAV infection. Neutralising antibody responses are crucial for protection against homotypic IAV infection, thus it is important to assess antibody functionality in addition to overall titre. Serum samples from γ-Flu-vaccinated and control mice were used to treat live A/PR8, and virus:serum mixtures were used to infect monolayers of MDCK cells. After a 22h incubation period, cells were stained with DAPI to visualise cell nuclei, and with murine anti-APR8 and FITC-conjugated anti-murine antibodies to visualise IAV-infected cells. Fluorescence levels of each fluorophore were quantified, and FITC-fluorescence relative to DAPI-fluorescence was calculated to determine the average IAV infectivity per cell. Quantified fluorescence of serum-treated virus samples were then compared to untreated virus only controls. As shown in **Figure 3B**, no reduction in infectivity was detected for virus treated with PBS-mock control sera, indicating that the murine sera from naïve animals had no effect on IAV infectivity. Conversely, infectivity was significantly reduced when A/PR8 was treated with serum from DI-, Ice- and RT-γ-Flu vaccinated mice. Interestingly, immune sera from mice vaccinated with Ice- and RT-γ-Flu was significantly more effective at neutralising A/PR8 when compared to immune sera from mice vaccinated with DI-γ-Flu. Representative images of virus neutralisation were also taken at a 1:10 serum dilution, and similarly demonstrate a clear reduction in foci for all γ-Flu groups, with antibodies induced by Ice- and RT-γ-Flu vaccination being the most effective (**Figure 3C**). This trend is likely due to the higher titre of total IgG present in immune sera from Ice- and RT-γ-Flu vaccinated animals, compared to those immunised with DI-γ-Flu.

**Figure 4:**
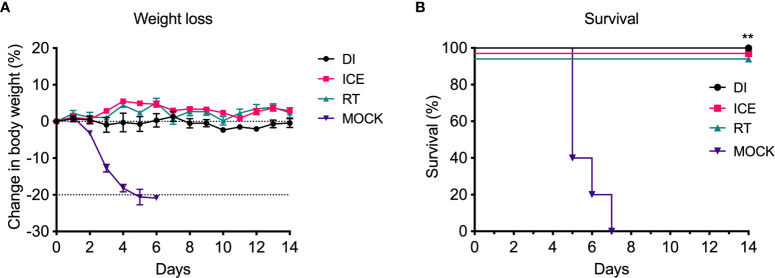
Vaccination with γ-Flu protects against lethal homotypic challenge. Mice were vaccinated intranasally with γ-Flu irradiated at different temperatures (DI, Ice and RT), or PBS-mock control. 21 days later, mice were intranasally challenged with a lethal dose of A/PR8. **(A)** Weight was monitored daily and a 20% loss of starting weight was considered as the humane endpoint (dotted line), at which point mice were euthanised. **(B)** Overall survival was plotted, and a two-tailed Fisher Exact test was used to determine statistical significance compared to the Mock control group (**P < 0.01, n = 5 mice per group).

Given the observed differences in functionality of γ-Flu-induced antibodies, we challenged vaccinated animals with live IAV to assess if these variations would translate to detectable differences in protective efficacy. Initially, the ability of DI-, Ice-, and RT-γ-Flu to mediate homotypic protection was investigated. Three weeks post-vaccination, mice were challenged with a lethal dose of homotypic A/PR8. No clinical symptoms were observed and no weight loss was recorded for all vaccinated groups, in contrast to PBS-mock control mice that succumbed to A/PR8 challenge and showed progressive weight loss to reach the humane end point of 20% body weight loss by day 7 post-infection (**Figure 4A**). Importantly, all vaccinated mice, irrespective of vaccine irradiation temperature, show 100% survival based on using 20% bodyweight loss as the humane end point (**Figure 4B**). This indicates that the antibody responses shown in **Figure 3**, though variable, were sufficient to induce robust homotypic protection.

**Figure 5:**
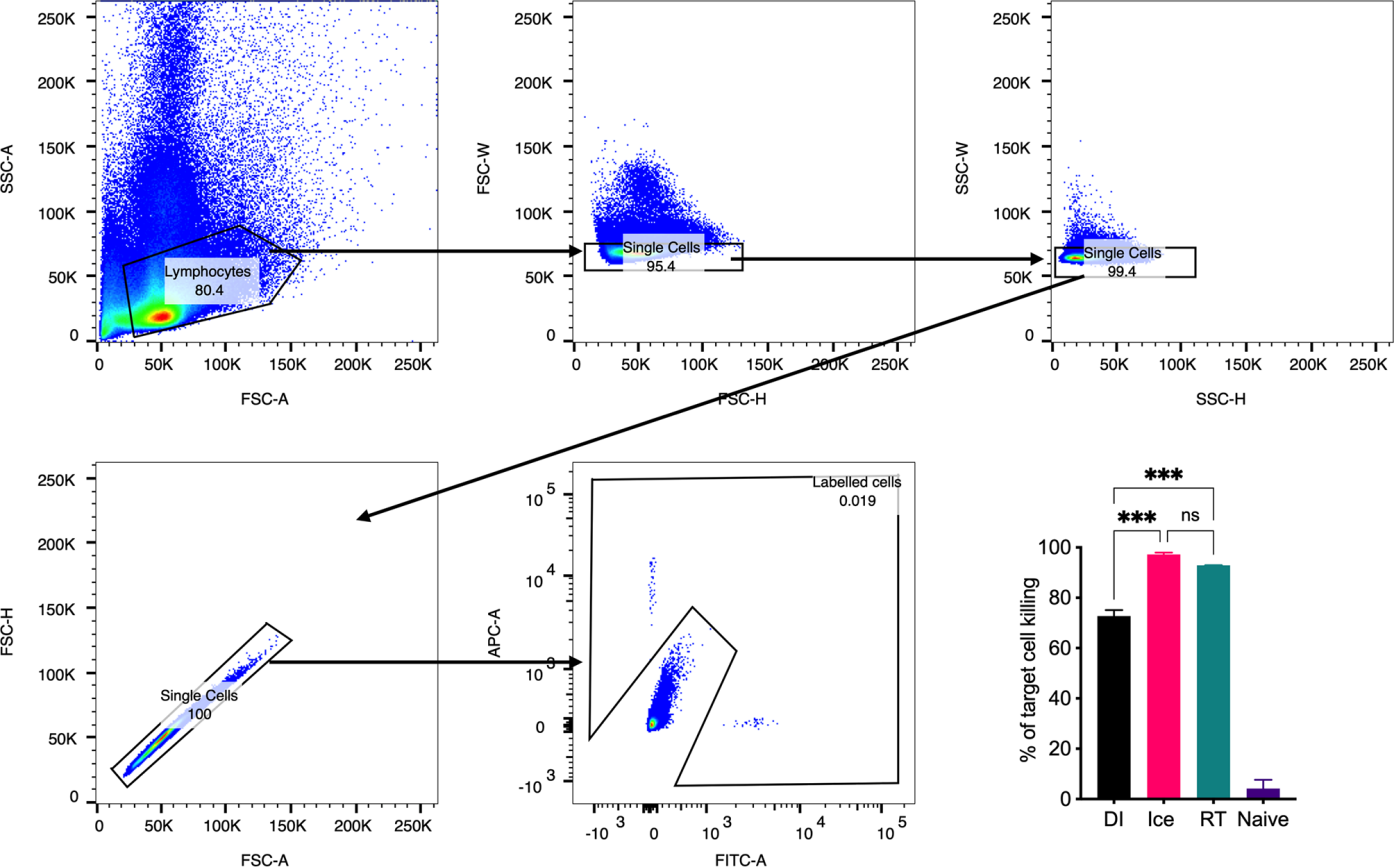
CTL responses are induced by vaccination with γ-Flu. Mice were vaccinated intravenously with γ-Flu preparations (RT-, Ice-, and DI-γ-Flu) or treated with PBS as mock control. 7 days later equal ratios of NPP-pulsed (CFSE labelled) and unpulsed (CTR labelled) splenocytes from naïve donor mice were injected into γ-Flu vaccinated mice or mock-vaccinated controls. 24 hours later splenocytes were harvested, processed and analysed using flow cytometry. Gating strategy is shown. The change in ratio of pulsed to unpulsed splenocytes after injection into vaccinated animals was used to calculate the percentage killing of pulsed cells. Data presented here as mean percentage +/- SEM and analysed using one-way ANOVA (***p < 0.001, ns, not significant, n = 3).

Importantly, a key feature of γ-Flu is its ability to induce cross-protective CD8^+^ T-cell responses against vaccine and non-vaccine IAV strains. To assess the effect of the differential irradiation temperatures on the induction of CD8^+^ T-cell responses, an *in vivo* CTL assay was performed. Here, the killing of IAV NPP-pulsed splenocytes (target cells) was assessed in vaccinated and non-vaccinated animals. NP has been identified as a key CD8^+^ T cell IAV antigen (25). As shown in **Figure 5**, splenocytes from naïve control mice show a 1:1 ratio of pulsed target cells to unpulsed cells, indicating no non-specific killing of targets cells *in vivo*. Conversely, we detected a substantial loss of NPP-pulsed cells relative to unpulsed cells in all three γ-Flu vaccinated groups. This demonstrates the ability of all γ-Flu preparations to induce a robust IAV-specific CTL responses as pulsed target cells were lysed within 24h of injection into immunised animals. Interestingly, animals vaccinated with Ice-γ-Flu and RT-γ-Flu showed significantly more effective CTL responses (97% and 93% killing of IAV-pulsed targets, respectively) compared to animals vaccinated with DI-γ-Flu (73% killing of IAV-pulsed targets).

**Figure 6:**
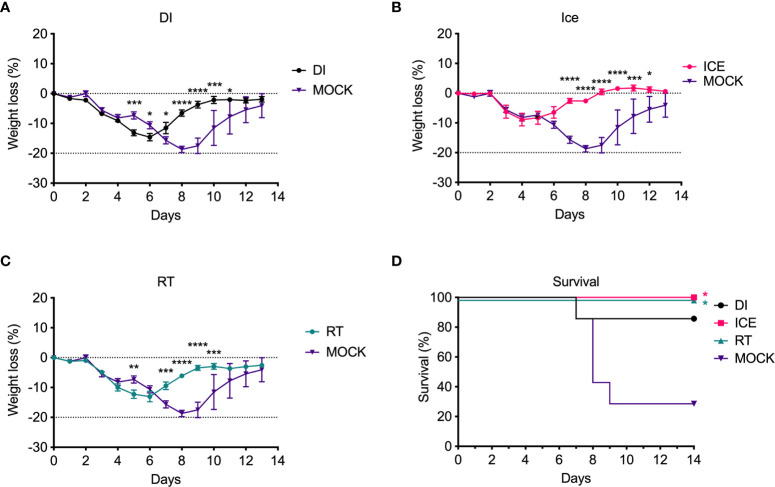
Vaccination with γ-Flu protects against lethal challenge with a drifted IAV strain. Mice were vaccinated intranasally with γ-Flu (γ-A/PR8 H1N1) irradiated at different temperatures, or PBS as mock control. 21 days later mice were challenged intranasally with a lethal dose of A/California H1N1. **(A-C)** Weight loss was measured daily, with a 20% loss of starting weight (dotted line) was considered as the humane end point. Weight loss was analysed by Two-Way ANOVA. **(D)** Survival rates were plotted, and a Two-Tailed Fisher-Exact test was used for analysis by comparing vaccinated groups to the PBS-MOCK vaccinated group (*p < 0.05, **p < 0.01, ***p < 0.001, ****p < 0.0001), n = 7 mice/group).

**Figure 7:**
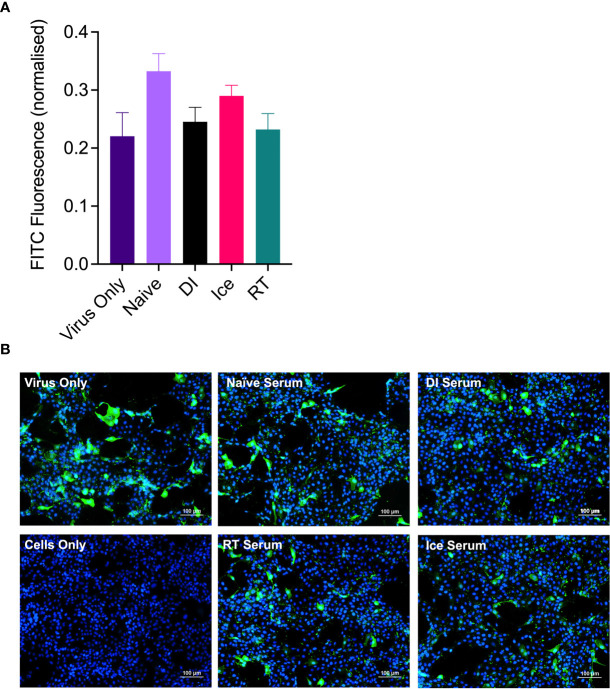
γ-Flu does not induce cross-neutralising antibody responses. Mice were vaccinated intranasally with γ-Flu (γ-A/PR8 H1N1) irradiated at room temperature (RT), on ice water (Ice) or on dry ice (DI). 20 days post-vaccination immune serum was harvested and the ability to neutralise A/California was measured by focus-forming inhibition assay. Live A/California was treated with pooled serum samples from the three vaccine groups or with serum from mock-vaccinated mice. Virus + serum mixtures were used to infect MDCK cell monolayers at MOI of 0.1. **(A)** FITC-fluorescence (green) indicative of A/California replication was measured relative to DAPI-fluorescence (blue), indicative of cell nuclei. **(B)** Representative images of cell monolayers showing A/California infection levels after pre-treatment with a 1:10 dilution of serum samples. Experiments were performed in triplicate and quantitative data was analysed by One-Way ANOVA. Data was not significant.

Enhanced IAV-specific CTL responses should theoretically translate to enhanced cross-protection against newly emerging IAV strains. To assess this, mice were vaccinated intranasally with different γ-Flu preparations (based on A/PR8 [H1N1]), or PBS-mock control. Three weeks later, mice were intranasally challenged with a lethal dose of A/California, the pdmH1N1 strain. As shown in **Figure 6**, all vaccinated and non-vaccinated mice experienced some weight loss following A/California infection, however mice vaccinated with Ice-γ-Flu showed less weight loss and faster recovery than the other vaccine groups. Furthermore, 100% survival was recorded for mice vaccinated with Ice-γ-Flu and RT-γ-Flu, whereas 86% survival occurred in mice vaccinated with DI-γ-Flu (1 out of 7 mice reached the humane end point of 20% weight loss). Overall, while γ-Flu vaccination was associated with significantly less weight loss and faster recovery time in all vaccinated groups, only Ice-γ-Flu and RT-γ-Flu was associated with significantly higher survival rates compared to the unvaccinated group. This outcome is consistent with the enhanced CTL responses (**Figure 5**). To rule out the possibility that the protection demonstrated in **Figure 6** was mediated by neutralising antibody responses, we tested the ability of serum generated by different γ-Flu preparations to neutralise A/California. Live A/California was treated with serial dilutions of serum generated by DI-, Ice- or RT-γ-Flu, or naïve serum as a control. Virus + serum was then added to confluent monolayers of MDCK cells and allowed to adhere for 2 hours before unbound virus was washed away. Cells were incubated for a further 2 hours at 37°C to allow virus growth then cells were fixed and stained with murine anti-A/California serum used as a primary antibody. As expected, we observed no cross-neutralisation generated by A/PR8 based γ-Flu preparations against A/California H1N1 (**Figure 7**).

## Discussion

IAV remains an important public health concern due to its high mutation rates and potential to cause global pandemics. Current vaccines only offer strain-specific protection due to the reliance on humoral immune responses against highly mutagenic HA and NA surface antigens rather than cross-protective responses against the conserved internal IAV components. We have developed an effective whole-IAV vaccine capable of protecting against multiple IAV strains and subtypes. For this vaccine candidate, IAV is inactivated using γ-irradiation (generating γ-Flu), and the heterosubtypic protection is specifically mediated by induction of cross-reactive cytotoxic T cell responses (5). While previous publications illustrated the underlying mechanisms for the cross-protective immunity, this study aims to improve the immunogenicity of γ-Flu by manipulating irradiation conditions.

Sterilisation of materials for biomedical analysis using γ-radiation is typically performed while the sample is frozen on dry ice to minimise structural damage. For example, serum samples from an Ebolavirus vaccine clinical trial were irradiated frozen at 50 kGy, and antibody binding detected by ELISA was well-maintained after this treatment (26). Bone allografts are also often sterilised whilst frozen, as bones are less brittle when irradiated on dry ice compared to irradiation with the same dose at room temperature (27). Our previous publications describing γ-Flu (2–5, 15, 28), γ-irradiated *Streptococcus pneumoniae* vaccine (γ-PN) (29), and a γ-irradiated rotavirus vaccine (30) all used irradiation on dry ice. Similarly, an experimental Venezuelan Equine Encephalitis Virus vaccine is also γ-irradiated while frozen on dry ice (31). Importantly, we have specifically advocated for DI-irradiation over RT-irradiation when using comparable high irradiation doses, as the use of frozen materials is associated with enhanced structural integrity and immunogenicity (15). However, previous studies did not investigate the immunogenicity of irradiated materials that received different sterilising doses relevant to different irradiation conditions.

It is well established that pathogens are more sensitive to inactivation by γ-irradiation at higher temperatures (17–19), which lowers the total sterilising dose required (12). In fact, this is the first study to consider the impact of irradiation temperature on the DS_SAL_ and directly compare the immunogenicity of sterile IAV preparations inactivated with different DS_SAL_ doses of γ-rays at different temperatures. Interestingly, our data show improved vaccine immunogenicity when using lower irradiation doses at higher temperatures. While previous studies have shown that more free radicals form and therefore more protein damage would occur when irradiating at higher temperatures (18), the lower dose of radiation required to reach the DS_SAL_ could explain the efficacy of Ice- and RT-γ-Flu. In fact, utilising these conditions would negate the need to keep samples frozen with an added advantage of a faster irradiation process.

To ensure that the heightened efficacy of ice and RT-irradiated samples was not due to residual live virus, sterility was confirmed for each preparation by three passages in MDCK cells. We have previously shown this method of sterility testing to be effective in detecting as little as 2 focus-forming units in a treated sample (30). **Figure 1** clearly shows all three preparations were free from viable virus over multiple passages. Furthermore, we used a very high MOI-equivalent of 600 to demonstrate sterility. Importantly, these data confirm that γ-Flu irradiated at sterilising doses does not have the ability to undergo recombination to produce viable virions.

We subsequently analysed the structural integrity of these sterilised γ-Flu samples by measuring HA and NA function. We found equivalent functionality for all preparations tested (**Figure 2**), which suggests that the γ-Flu preparations would be highly immunogenic due to retained function of key antigens. Furthermore, IFN-I specifically relies on the ability of IAV HA to bind to sialic acid receptors on IFN-I producing cells for virus internalisation (32). In fact, we have previously published the ability of γ-Flu to induce superior IFN-I responses compared to commercial IAV vaccines and demonstrated IFN-1-dependent T cell activation (28).

Of interest, current inactivated IAV vaccines induce antibodies of a narrow breadth, whereas responses to natural IAV infection include a small population of broadly neutralising antibodies against the HA stalk (33), an area that is highly conserved (34). However, antibodies to the HA stalk may still be overcome by mutations (35). We initially tested the effect of irradiation temperature on the ability of γ-Flu to induce neutralising antibody responses and homotypic protection. Interestingly, while all γ-Flu preparations induced strong A/PR8-specific IgG and neutralising responses, Ice-γ-Flu and RT-γ-Flu performed better than DI-γ-Flu (**Figure 3**). Nonetheless, all γ-Flu preparations induced complete protection against homotypic A/PR8 challenge (**Figure 4**).

We found that Ice-γ-Flu and RT-γ-Flu also outperformed DI-γ-Flu for induction of CTL responses (**Figure 5**), and protection against lethal drifted challenge (**Figure 6**). It is well established that live IAV-induces CTL responses that can target the conserved internal NP, matrix and polymerase proteins (36, 37). Our previous work has illustrated that antibodies induced by γ-Flu are strain-specific (3), and that cross-protection arises through cell-mediated responses (5). In the present study we confirm that antibodies generated against all three γ-Flu preparations were unable to neutralise the drifted pdmH1N1 (**Figure 7**), and so protection demonstrated in **Figure 6** is expected to be mediated by the enhanced CTL responses (**Figure 5**).

The reduced efficacy of DI-γ-Flu compared to RT- and ice-irradiated preparations suggest that irradiating frozen materials using high dose may not be the best approach to minimise the damage to viral proteins. Instead, a balanced irradiation process that includes the use of low doses of γ-rays to inactivate unfrozen materials at cold or RT conditions could be utilised to produce highly immunogenic vaccine preparations. Indeed, Cote et al. (38) showed that the irradiation conditions of anthrax spores could be adjusted to meet a SAL of 10^-6^ using room temperature or ice-irradiation while maintaining the biological structure required for biomedical testing. This change in irradiation conditions could overcome biosecurity issues associated with the inadvertent release of live anthrax spores by the US Department of Defense (39). A radiation-attenuated malaria vaccine PfSPZ is reported to receive a low dose of γ-irradiation at RT prior to harvesting the sporozoites from the mosquito (40).

Recently, electron beam (eBeam) irradiation has been employed as an alternative to γ-irradiation. eBeam has several advantages over γ-irradiation including significantly higher dose rates and safety (41). Importantly, our findings demonstrate that liquid samples can be highly immunogenic when irradiating to the DS_SAL_ compared to frozen samples which is expected to simplify manufacturing procedures for irradiated vaccines regardless of the technology used. In fact, these findings may also support the use of eBeam in vaccine development.

In this study, precise calculation of the DS_SAL_ allowed us to prepare highly immunogenic γ-Flu using substantially lower dose of irradiation while maintaining internationally acceptable level of sterility. These data also indicate that ice or RT-irradiation is far less damaging than previously thought if the concept of the DS_SAL_ is properly applied. These observations offer new and improved insights into the use of γ-irradiation to inactivate viruses for vaccine purposes and they could be utilised to vastly improve the feasibility of scale-up manufacturing.

## Data Availability Statement

The original contributions presented in this study are available by request to mohammed.alsharifi@adelaide.edu.au.

## Ethics Statement

The animal study was reviewed and approved by the Animal Ethics Committee, University of Adelaide.

## Author Contributions

MA and ES conceived and designed the study. ES, CG, SD, and JD performed experiments. ES wrote the manuscript. SD, TH, JD, and MA assisted in experimental design and preparation of the manuscript. MA and JD supervised the study. All authors contributed to the article and approved the submitted version.

## Funding

This work was supported by the following funding sources, an Australian Institute of Nuclear Science and Engineering (AINSE) Research Award (ALNGRA15517 to MA) and an Australian Government Research Training Program (RTP) Scholarship (to ES).

## Conflict of Interest

MA is head of the Vaccine Research Group at the University of Adelaide and the Chief Scientific Officer of Gamma Vaccines Pty Ltd and TH is the Executive Chairman of Gamma Vaccines Pty Ltd. This does not alter adherence to policies on sharing data and materials. Gamma Vaccines Pty Ltd has no role in the study design, data collection and analysis, decision to publish, and preparation of the manuscript.

The remaining authors declare that the research was conducted in the absence of any commercial or financial relationships that could be construed as a potential conflict of interest.

## Publisher’s Note

All claims expressed in this article are solely those of the authors and do not necessarily represent those of their affiliated organizations, or those of the publisher, the editors and the reviewers. Any product that may be evaluated in this article, or claim that may be made by its manufacturer, is not guaranteed or endorsed by the publisher.
